# The Risk of Exacerbation of Myasthenia Gravis After COVID‐19 Omicron Infection

**DOI:** 10.1002/brb3.70074

**Published:** 2024-10-20

**Authors:** Nana Zhang, Ziya Wang, Dongren Sun, Hongxi Chen, Hongyu Zhou

**Affiliations:** ^1^ Department of Neurology, West China Hospital Sichuan University Chengdu Sichuan Province PR China

**Keywords:** COVID‐19, myasthenia gravis, Omicron

## Abstract

**Objective:**

The aim of this study is to ascertain whether COVID‐19 Omicron infection is associated with exacerbations in these myasthenia gravis (MG) patients.

**Result:**

In total, 289 MG patients (comprising 60% females, with an average age of 46 ± 15 years) were enrolled. A total of 80.9% of MG patients reported a COVID‐19 infection, with the majority experiencing a benign course (88%). MG patients who experienced COVID‐19 infection demonstrated a higher likelihood of MG exacerbation, compared to those without the infection (18.8% vs. 7.3%, *p* = 0.039). In the survival analysis, after adjusting for confounding factors, the hazard ratio (HR) for exacerbation post‐infection was found to be 3.38 (95% CI 1.20–9.53, *p* = 0.021). Compared to the exacerbation rates observed in JTA21, an increase was noted in DTM23 among COVID‐19‐infected MG patients (4.4% vs. 17.2%, *p* < 0.001).

**Conclusion:**

The COVID‐19 is the risk of MG exacerbation.

## Graphical abstract

This retrospective cohort study employed questionnaires to investigate the COVID‐19 infection status and exacerbation of myasthenia gravis (MG) patients after the relaxation of prevention and control measures in China. Statistical results demonstrate that COVID‐19 infection poses a significant risk for MG exacerbation, compared to the MG group without COVID‐19.

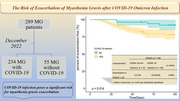

## 1 Background

Myasthenia gravis (MG) is an autoimmune disease characterized by the presence of various antibodies at the neuromuscular junction. The predominant symptoms are characterized by fatigability and fluctuating muscle weakness, primarily affecting the bulbar, limb, and respiratory muscles (Verschuuren et al. 2022). Infections are known to play a significant role in MG exacerbation and crisis, as commonly reported by MG patients experiencing acute deterioration or exacerbation (Jakubíková et al. 2021; Gilhus et al. 2019; Nelke et al. 2022).

Coronavirus disease 2019 (COVID‐19), caused by severe acute respiratory syndrome coronavirus 2 (SARS‐CoV‐2), can lead to pneumonia and acute respiratory distress and is characterized by asymptomatic and pre‐symptomatic transmission (Verschuuren et al. 2022). SARS‐CoV‐2 spreads primarily via short‐range airborne aerosol, respiratory droplets, and contact with infectious respiratory droplets (To et al. 2021). The respiratory samples of individuals who had been exposed to SARS‐CoV‐2‐infected patients tested positive in real‐time polymerase chain reaction (RT‐PCR) analysis a day before symptom onset, suggesting that asymptomatic individuals can be infectious (Pan et al. 2020; Y. Li et al. 2021). During the COVID‐19 pandemic, MG patients experiencing bulbar and respiratory weakness, and undergoing immunosuppressive therapy, were likely to be in high‐risk categories for SARS‐CoV‐2 infection and severe COVID‐19 disease progression (Salik, Rodhouse, and Barst 2020; Guidon and Amato 2020). Contact with COVID‐19‐infected individuals has been proven to be significantly associated with COVID‐19 infection in MG patients (de León et al. 2023).

A systematic review has revealed that SARS‐CoV‐2 may potentially trigger a wide range of autoimmune and rheumatic manifestations (Tang, Hsu, and Chen 2021). The risk of immune‐mediated neurological events is increased in individuals with SARS‐CoV‐2 infection as evidenced by a population‐based study (X. Li et al. 2022). In December 2022, China experienced a large‐scale epidemic of the COVID‐19 Omicron variant over a short period. Given the context of the COVID‐19 pandemic, understanding the correlation between COVID‐19 infection and MG exacerbation is crucial for the management of high‐risk patients. However, prior studies have yielded varied conclusions regarding this issue (Muppidi et al. 2020; Stascheit et al. 2023; Karimi et al. 2022; Businaro et al. 2021; Solé et al. 2021).

In this observational study, we employed questionnaires or telephone contact to investigate the infection status of MG patients in our center's database and to evaluate their disease status. Simultaneously, previous clinical data of these patients were retrospectively retrieved. The aim of this study is to ascertain whether COVID‐19 Omicron infection is associated with exacerbations in these MG patients.

## 2 Materials and Methods

### 2.1 Study Design and Participants

This retrospective study analyzed data from patients in the cohort at the Medical Center of Southwest China (Department of Neurology, West China Hospital, Sichuan University, China) spanning January 2016 to January 2022. MG patients were included in this study based on the following criteria: For diagnosing MG, the primary criterion involves typical symptoms such as partial or generalized striated muscle fatigue, which worsen after exercise and improve with rest. Additionally, a definitive diagnosis of MG required a positive serologic test for autoantibodies such as acetylcholine receptor or muscle‐specific tyrosine kinase autoantibodies and/or a positive neostigmine test and/or abnormal repetitive nerve stimulation, and the exclusion of other neuromuscular junction diseases (Gilhus 2016). Patients with invalid questionnaires, incomplete clinical information, or concurrent infections such as influenza were excluded. Demographic and clinical data were collected and followed up biannually through routine clinical visits, including assessments with the Myasthenia Gravis Activities of Daily Living (MG‐ADL) scale and the Myasthenia Gravis Foundation of America (MGFA) classification (Kong et al. 2022).

### 2.2 Questionnaire Design and Data Collection

We designed an online questionnaire. The questionnaire comprises 27 questions, with detailed information provided in the Supporting Information. Information on COVID‐19 infections and MG exacerbations during the COVID‐19 pandemic was collected from December 1, 2022, to March 10, 2023 (DTM23), by randomly selecting 500 patients from the MG database and inviting them to complete the questionnaires via online delivery and telephone contact.

After the relaxation of prevention and control measures in December 2022, a substantial COVID‐19 outbreak ensued, affecting over 82% of China's population, with some infected individuals not undergoing nucleic acid or antigen testing (Fu et al. 2023). Prior studies revealed the face‐to‐face contact with a COVID‐19‐infected individual was a notable risk factor for COVID‐19 infection, particularly in MG patients (de León et al. 2023; Ricciardi et al. 2021). Therefore, in our study, COVID‐19 infection was defined if the patient met at least one of the following criteria: (1) typical symptoms like fever, cough, or sore throat or contact with confirmed SARS‐CoV‐2 infected household members between December 1, 2022, and March 10, 2023 (de León et al. 2023); (2) a positive nasopharyngeal swab RT‐PCR test; or (3) positive serologic tests for SARS‐CoV‐2. MG exacerbation was defined as a deterioration of at least 2 points on the Activity of Daily Living (ADL) scale, compared to the last visit (Jakubíková et al. 2021; Garibaldi, Siciliano, and Antonini 2021). The severity of COVID‐19 was classified using a 5‐point scale: 1 for *asymptomatic* *condition and history of exposure to SARS‐CoV‐2‐infected patients*, 2 for *mild symptoms like fever or headache*, 3 for *COVID‐19 pneumonia requiring home care*, and 4 for *hospitalization*, and 5 for *COVID‐19‐associated ICU admission*.

The demographic and clinical characteristics of the participants, including sex, age, disease duration, thymectomy, and comorbidities, were retrospectively reviewed. Participants with invalid questionnaires or incomplete data were excluded from the study.

To further investigate the impact of COVID‐19 on MG patients, those with an onset prior to March 2021 were selected from the enrolled cohort. Exacerbation and treatment records of these MG patients, spanning January 2021 to April 2021 (JTA21), were extracted from our database to compare the exacerbations in DTM23 and JTA21 within the same patients. MG treatment was categorized using a 4‐point scale: 0 for *pyridostigmine or no treatment*, 1 for *glucocorticoid*, 2 for *nonsteroidal immunosuppressants*, and 3 for *glucocorticoid and nonsteroidal immunosuppressants*. If the patient's MG treatment regimen remains unchanged throughout JTA21 and DTM23, it is deemed constant.

### 2.3 Statistical Analysis

Continuous variables were presented as mean ± standard deviation (SD) or, if not normally distributed (verified by the Kolmogorov–Smirnov test), as median and interquartile range (IQR). Categorical variables were described as frequencies and percentages. Mean differences between the two groups were analyzed using unpaired *t*‐tests, while median differences were evaluated with Mann–Whitney U tests. Categorical variables were compared using Fisher's exact test.

Kaplan–Meier curves were generated to depict the time to MG exacerbation, with differences between the two groups assessed using a log‐rank test. We designed December 1, 2022, as time zero, with the endpoint defined as the date of the first exacerbation during DTM23. For relapse‐free patients, the endpoint was defined as the date they completed the questionnaire. Univariable Cox regression analysis was performed to calculate the hazard ratio (HR).

The Change‐in‐Estimate approach was utilized in the univariable Cox regression analysis to select confounding factors identified a priori through literature review. Variables were retained in the adjusted model if their removal resulted in a change in the HR of 10% or greater. The final selected variables were incorporated in a multivariate Cox regression analysis.

The multivariate Cox regression analysis was utilized to assess the impact of COVID‐19 infection on MG exacerbation, with a Schoenfeld residual test conducted to evaluate potential violations of the proportional hazards assumption.

McNemar's test was utilized to compare the exacerbations and treatment between DTM23 and JTA21 periods in the JTA21 patients.

All statistical analyses were conducted using RStudio, version 4.2.1, with statistical significance set at *p* < 0.05.

### 2.4 Standard Protocol Approvals and Patients’ Consent

This study received approval from the Medical Ethics Committee of West China Hospital, Sichuan University (Approval No. 2019(536)). All patients included in the database at the Medical Center of Southwest China had signed informed consent forms upon entry for follow‐up. At the outset of the questionnaire, the investigator provided patients with comprehensive information regarding the study's purpose and the confidentiality of their personal information and subsequently invited them to voluntarily complete the questionnaire.

## 3 Results

### 3.1 Study Population

**FIGURE 1:**
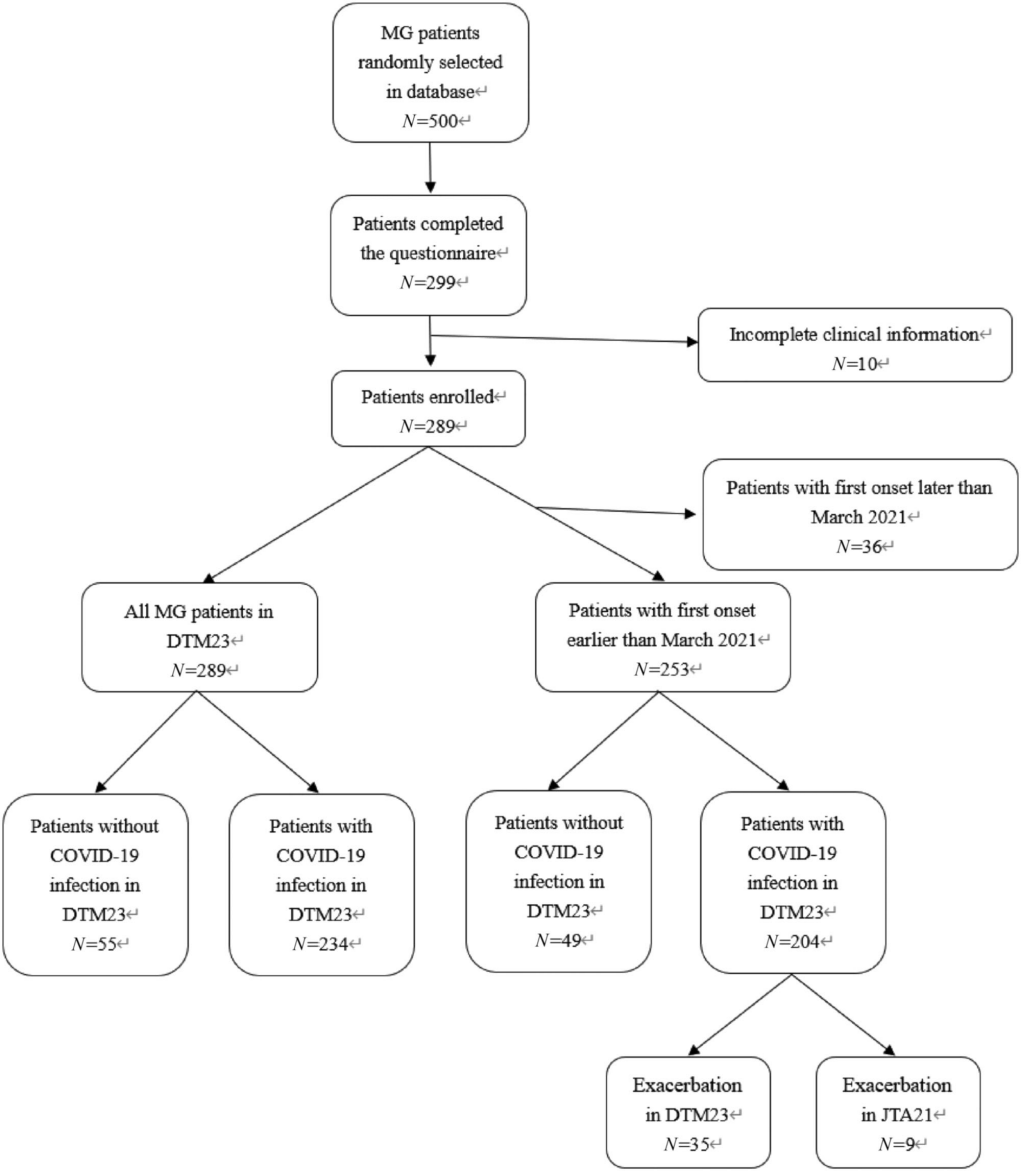
Study flow chart.

We randomly selected 500 patients from our MG database and invited them to complete the questionnaires through online delivery and telephone contact. We reclaimed 299 valid questionnaires. Of the 299 valid questionnaires retrieved, 10 were completed by patients with incomplete clinical data. The response rate was 59.8%, and a study flow chart is depicted in Figure 1.

### 3.2 Demographic and Clinical Characteristics

In total, 289 MG patients validly completed the questionnaire, among whom 173 (60%) were female. The cohort predominantly comprised younger patients, with a median age of 47 years. Among the cohort, 69 patients (24%) had comorbidities, including hyperthyroidism, coronary heart disease, hypertension, and diabetes. Of the 48 MG patients with exacerbation, 13 patients (27%) had comorbidities, whereas 56 patients (23%) had comorbidities in the remaining 241 patients. There were no statistically significant differences between the two groups (*p* = 0.568). The demographic and clinical characteristics of the recruited patients are detailed in Table 1.

**TABLE 1:** Demographic and clinical characteristics of MG patients.

Characteristics	DTM23, *N* = 289		
	COVID‐19 non‐infection, *N* = 55	COVID‐19 infection, *N* = 234	*p*‐value^a^
Exacerbation in DTM23, *n* (%)	4 (7.3%)	44 (18.8%)	0.039^*^
Sex (female), *n* (%)	26 (47%)	147 (63%)	0.034^*^
Age, median (IQR), years	50 (36, 58)	47 (34, 56)	0.351
MG duration, median (IQR), years	6.0 (4.0, 11.0)	5.0 (3.0, 9.0)	0.254
Thymectomy, *n* (%)	17 (31%)	67 (29%)	0.738
Antibody			0.887
Achr	38 (69%)	167 (71%)	
Musk	3 (5.5%)	15 (6.4%)	
Double‐negative	10 (18%)	33 (14%)	
Other	4 (7.3%)	19 (8.1%)	
Comorbidities	10 (18.2%)	59 (25.2%)	0.355
MGFA class ≥ III at last visit, *n* (%)	5 (9.1%)	24 (10%)	0.796
MG treatment prior to COVID‐19, *n* (%)			0.009^*^
Pyridostigmine or no treatment	28 (51%)	67 (29%)	
Glucocorticoid	16 (29%)	74 (32%)	
Nonsteroidal immunosuppressants	6 (11%)	51 (22%)	
Glucocorticoid and nonsteroidal immunosuppressants	5 (9.1%)	42 (18%)	
COVID‐19 vaccine, *n* (%)	28 (51%)	134 (57%)	0.393
Severity of COVID‐19, *n* (%)			
1		15 (6.4%)	
2		188 (80%)	
3		8 (3.4%)	
4		20 (8.5%)	
5		3 (1.3%)	

Abbreviations: ADL = Activities of Daily Living; MGFA = Myasthenia Gravis Foundation of America; SD = standard deviation.

^a^ Pearson's Chi‐squared test; Wilcoxon rank sum test; Fisher's exact test.

* *p* < 0.05 was statistically significant.

MG Patients who were treated with corticosteroids or immunosuppressants experienced a higher incidence of COVID‐19 infections. No significant difference was observed in the rates of COVID‐19 vaccination between infected and non‐infected patients with MG.

Out of 234 COVID‐19‐infected MG patients, 147 were female (63%) and 87 were male (37%). The majority of COVID‐19 infections were of a benign course, classified between Points 1 to 3. The most common symptoms were respiratory (117 patients, 50%) and systemic discomfort (174 patients, 74.4%). One patient was concurrently diagnosed with myocarditis and pneumonia. Eight COVID‐19‐infected MG patients (3.4%) experienced respiratory failure requiring mechanical ventilation, of whom three were admitted to the ICU. According to the responses, no patients died of the COVID‐19 infection. Post‐COVID‐19 infection, 35 patients (14.9%) reported symptoms of fatigue, tiredness, and shortness of breath. The median time from infection to investigation was 48 days, and the IQR was 41 to 69 days.

Among the 289 patients, 48 experienced an exacerbation during DTM23. The median duration from first exacerbation to investigation was 39 days, with an IQR of 20 to 49 days. Among MG patients infected with COVID‐19, the median duration from infection to first exacerbation was 14 days, with an IQR of 3 to 24 days. Data on the exacerbation of 48 MG patients in DTM23 are detailed in Table 2.

**TABLE 2:** Exacerbation of MG patients in DTM23.

	Exacerbation patients, *N* = 48	
Exacerbation	No COVID‐19 infection, *N* = 4	COVID‐19 infection, *N* = 44
Change in ADL, *n*		
2–4	3	27
4–6	0	6
6–8	1	7
8–10	0	4
Ventilator, *n*	0	4
Gastric tube, *n*	0	2

Abbreviation: ADL = Activities of Daily Living.

### 3.3 MG Exacerbation Associated With COVID‐19 Infection

**FIGURE 2:**
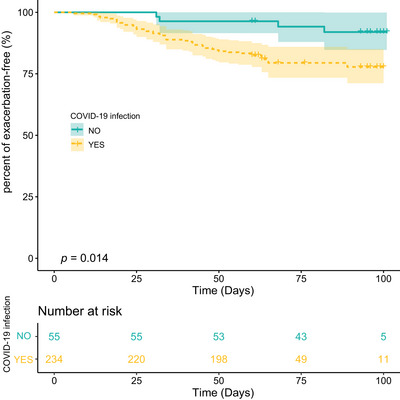
The Kaplan–Meier curves for MG patients. Log‐rank test was applied to compare the exacerbation rate between MG patients with and without the COVID‐19 infection during DTM23.

MG patients with COVID‐19 infection exhibited a higher likelihood of exacerbation, compared to those without COVID‐19 infection (18.8% vs. 7.3%, *p* = 0.039, Table 1). Kaplan–Meier graphs were utilized to plot the occurrence of MG exacerbation in DTM23, comparing the COVID‐19‐infected and non‐infected groups. A statistically significant difference was observed in the Kaplan–Meier curves between the two groups (*p* = 0.014, Figure 2). In the univariate Cox regression analysis, the HR for the first exacerbation post infection was 3.38 (95% CI 1.20–9.53, *p* = 0.021). Change‐in‐Estimate approach indicated that the established confounders did not alter the main result (Figure S1). Additionally, a multivariate Cox regression analysis was conducted as a sensitivity analysis, yielding results consistent with the main analysis (Figure S2).

### 3.4 MG Exacerbation of JTA21 Patients in DTM23 and JTA21

From the 289 enrolled MG patients, 253 individuals whose first onset occurred prior to March 2021 were selected. Exacerbation and treatment records of these MG patients, spanning January 2021 to April 2021 (JTA21), were retrieved from our database.

Among these patients, 204 (80.6%) reported a COVID‐19 infection in DTM23. Exacerbation characteristics for these patients in DTM23 and JTA21 are depicted in Table 3. In comparison to the JTA21 period, MG exacerbations increased following COVID‐19 infection in DTM23 (4.4% vs. 17.2%, *p* < 0.001). No similar differences were observed in the rest of the 49 non‐infected patients (4.1% vs. 8.2%, *p* = 0.687). Comparison of MG treatment among the 204 patients in DTM23 and JTA21 is detailed in Table S1. From the 204 patients, we selected COVID‐19‐infected patients whose treatment remained constant from JTA21 to DTM23 and compared their exacerbations between JTA21 and DTM23 using McNemar's test. The results indicated that exacerbations occurred more frequently after the infection in the same patients (*p* = 0.001, Table S2).

**TABLE 3:** Exacerbation characteristics of selected infected patients in DTM23 and JTA21.

	Selected infected patients, *N* = 204	
Exacerbation	DTM23, *N* = 35	JTA21, *N* = 9
Change in ADL, *n*		
2–4	20	7
4–6	5	2
6–8	7	0
8–10	3	0
Ventilator, *n*	2	0
Gastric tube, *n*	1	0

Abbreviation: ADL = Activities of Daily Living.

## 4 Discussion

Infection is a common trigger for exacerbation or crisis in MG (Gilhus et al. 2018; Gilhus 2023; Farina et al. 2022). Exacerbations of the disease can impose a significant burden on MG patients (Nelke et al. 2022). COVID‐19, an infectious disease caused by SARS‐CoV‐2, is characterized by rapid evolution and widespread transmission (Y. Li et al. 2021). This cohort study is aimed to investigate the effect of SARS‐CoV‐2 infection on exacerbations of MG.

Our study confirms COVID‐19 infection as a risk factor for MG exacerbation. In the study, the MG group with COVID‐19 exhibited a higher likelihood of exacerbation, compared to the MG group without COVID‐19 during the DTM23 period. The results offer clinical evidence supporting the notion that SARS‐CoV‐2 infection elevates the risk of MG exacerbation.

To mitigate confounding variables such as seasonal variations and differences in drug treatments among patients, we performed a comparison focusing on patients who contracted COVID‐19 in DTM23 but were not infected during JTA21. The findings consistently demonstrated the influence of COVID‐19 infection on MG exacerbations.

Infection, particularly respiratory tract infection, was common and potential for symptom exacerbation in MG patients. In a Canadian MG cohort conducted before the global COVID‐19 outbreak, the risk of infection in patients with MG was found to be twice that of a matched control group (Kassardjian et al. 2020). Reviewing the NMD‐ES registry in Spanish, infection emerged as the most closely associated risk factor for MG patients experiencing life‐threatening events, in agreement with other studies (Gilhus et al. 2018; Ramos‐Fransi et al. 2015; Nelke et al. 2022). Among the most common infections, respiratory tract infections, including influenza virus and *Mycoplasma pneumoniae*, were frequently cited as the primary cause of MG exacerbation by patients and hospital data (Gilhus 2023; Pscheidt et al. 2021; Blum et al. 2015; Iwasa et al. 2018). A multicenter retrospective study from Korea suggested that influenza infection is the sole independent factor for MG symptom exacerbation after adjusting for the confounding factors (Seok et al. 2017). Likewise, COVID‐19 infection likely posed a risk factor for exacerbation in MG patients within our cohort.

Recent data from the COVID‐19 Associated Risks and Effects in Myasthenia Gravis (CARE‐MG) registry indicate a concerning trend, where MG patients infected with COVID‐19 are more frequently hospitalized, experience increased disease exacerbations, and exhibit higher mortality rates, compared to the general COVID‐19 population, as corroborated by other studies (Muppidi et al. 2020; Stascheit et al. 2023; Karimi et al. 2022). Our study corroborates the notion that COVID‐19 infection is a risk factor for MG exacerbation as evidenced by both vertical and horizontal analyses comparing infected and non‐infected patients. Conversely, Businaro et al. and Solé et al. have posited the contrary view that COVID‐19 has minimal impact on the course of MG (Businaro et al. 2021; Solé et al. 2021). In contrast to our findings, Businaro et al.’s study, which included a cohort of 11 MG patients with COVID‐19 and 151 without the infection, did not observe a significant impact of the infection on MG exacerbation (Businaro et al. 2021). Similarly, in a registry‐based cohort study of MG patients, only 0.96% of whom had COVID‐19, no association was found between the severity of COVID‐19 and MG exacerbation as per the univariate analysis (Solé et al. 2021). IL‐6 and TNF‐*α*, which are associated with MG disease severity, were deregulated in COVID‐19 patients during the acute stage or early recovery (Tüzün, Huda, and Christadoss 2011; Schultheiß et al. 2022; Ramasamy and Subbian 2021). A prospective study also found IL‐6 upregulation in MG patients with COVID‐19 (Huan et al. 2024). However, the specific mechanisms of exacerbation after COVID‐19 infection remain to be further studied.

Increased susceptibility to infections is a well‐documented phenomenon in many autoimmune disorders, with immunosuppressive therapy identified as a contributing factor (Gilhus et al. 2018). Our observations align with this, as MG patients who had been taking corticosteroids or immunosuppressants, whether regularly or irregularly, prior to DTM23, exhibited an increased risk of SARS‐CoV‐2 infection, compared to those on no medication or solely cholinesterase inhibitors.

The course of COVID‐19 disease was predominantly benign, characterized by low severity and mortality rates. During DTM23, a significant COVID‐19 outbreak in December 2022 led to over 82% of China's population reporting infection as indicated by a four‐wave online survey (Fu et al. 2023). From December 1, 2022, to April 27, 2023, all confirmed SARS‐CoV‐2 genome sequences reported across the nation were identified as belonging to the Omicron variants (COVID‐19 Clinical and Surveillance Data n.d.). The enrolled patients with MG and COVID‐19 were very likely infected with the Omicron variants. In comparison to the Delta and Beta variants, the Omicron variant is associated with lower infection severity, a younger patient demographic, and higher transmissibility and infectivity (N. Yang et al. 2022; W. Yang et al. 2022; Meo et al. 2021; Madhi et al. 2022, Leiner et al. 2022), which aligns with the findings of our study indicating a benign infection course.

In this cohort study, no statistically significant differences were observed in COVID‐19 vaccination rates between infected and uninfected MG patients. As of April 2023, 90.6% of China's entire population had completed the primary series of COVID‐19 vaccinations (COVID‐19 Clinical and Surveillance Data n.d.). In early 2021, the vast majority of people received the SARS‐CoV‐2 inactivated vaccine, which was based on the original SARS‐CoV‐2 strain (H. Y. Li et al. 2022). However, the Omicron variants, characterized by complex mutations, can evade neutralization by most identified anti‐SARS‐CoV‐2 antibodies (Gao et al. 2023). Consequently, COVID‐19 vaccinations do not offer complete protection against infection with various COVID‐19 variants (Cheung, Chan, and Jin 2022; Zhang, Zhang, and Chen 2022), aligning with our study findings. Developing new and precise protective vaccines would be particularly beneficial for MG patients, especially those on immunosuppressive therapy.

Notably, our cohort was characterized by a younger age demographic. The median age was 47 years old. Previous research has indicated that older age is a risk factor for the exacerbation or worsening of MG (Alshekhlee et al. 2009). In the typical course of MG, older patients are more susceptible to exacerbations than their younger counterparts. Studies have revealed a strong association between viral burden and old age, with pre‐existing neurological conditions potentially worsening post‐COVID‐19, particularly in older individuals (Dewanjee et al. 2021). Furthermore, a cohort study has identified older age as a significant risk factor for severe COVID‐19 progression in MG patients (Jakubíková et al. 2021). Age is a pertinent factor in assessing the risk of MG exacerbation and severe SARS‐CoV‐2 infection. Although there was no statistically significant difference in age between the COVID‐19‐infected and non‐infected groups in our cohort, our findings among MG patients with a median age of 47 years underscore the importance of vigilant attention to exacerbation and medical management during COVID‐19, particularly in older MG patients.

There were some limitations in the study. First, this was a single‐center study. Factors such as age, education level, and acceptance of electronic questionnaires affected the survey participants, leading to selection bias. Moreover, the cross‐sectional nature of the survey and its relatively low response rate may have led to deviations from the actual number of infections or the true post‐infection conditions. Additionally, the inclusion of infected patients was based not strictly on laboratory tests but rather on typical COVID‐19 symptoms. The actual number of MG patients confirmedly infected with SARS‐CoV‐2 might be lower than those included in the study, but evidence suggests an increased likelihood of COVID‐19 in MG patients following close contact with individuals diagnosed via laboratory tests (de León et al. 2023), prompting the inclusion of cases based on contact history and clinical diagnosis.

In conclusion, this cohort study demonstrates that COVID‐19 infection poses a significant risk for MG exacerbation. The development of timely and targeted vaccines could greatly benefit MG patients.

## Author Contributions

**Nana Zhang**: investigation, visualization, data curation, writing–original draft. **Ziya Wang**: investigation, writing–original draft, visualization, data curation. **Dongren Sun**: methodology, visualization. **Hongxi Chen**: methodology, visualization. **Hongyu Zhou**: conceptualization, writing–review&editing, funding acquisition, supervision.

## Conflicts of Interest

The authors declare no conflicts of interest.

### Peer Review

The peer review history for this article is available at https://publons.com/publon/10.1002/brb3.70074.

## Supporting information



Supporting Information

FIGURE S1 Change‐in‐Estimate result.

FIGURE S2 The multivariate Cox regression analysis result.

TABLE S1. The treatment of 20 JTA21 patients.

TABLE S2 The JTA21 patients whose treatment is constant.

## Data Availability

The data supporting the findings of this study are available on request from the corresponding author. The data are not publicly available due to privacy or ethical restrictions.
