# Supplementary material for: The Risk of Exacerbation of Myasthenia Gravis After COVID‐19 Omicron Infection
Source: Brain Behav. 2024 Oct 20;14(10):e70074. doi: 10.1002/brb3.70074 (PMC11491296; doi:10.1002/brb3.70074)
Supplement: Supplementary file 1 — Supporting Information [file BRB3-14-e70074-s001.docx]

1. what is your name？
2. Current COVID-19 vaccination status

No, not vaccinated yet.

Yes, received 1 dose of COVID-19 vaccine

Yes, received 2 doses of COVID-19 vaccine

Yes, received 3 doses of COVID-19 vaccine

Yes, received 4 doses of COVID-19 vaccine

1. Have you been infected with the COVID-19?

At least one of the following conditions: (1) fever, sore throat, or other typical symptoms; (2) positive serologic tests for SARS‐CoV‐2; (3) positive nasopharyngeal swab RT-PCR testing; (4) contact with confirmed SARS‐CoV‐2 infected individuals (such as co-living members, colleagues, etc.)

NO

YES

1. Date of COVID-19 infection

If you confirmed the infection through a nasopharyngeal swab RT-PCR testing or serologic tests, fill in the date of the positive test; If no tests have been performed, fill in the date on which the symptoms appeared.

5. How severe do you feel your infection?

Asymptomatic infection

Mild

Moderate

Severe

6. what are your symptoms of infection

Fever, fatigue, chills

Nasal congestion, runny nose, sore throat, dry cough, difficulty breathing

Loss of smell, loss of taste

Chest tightness, palpitations

Abdominal pain, diarrhea, vomiting, loss of appetite

Dizziness, headache

Other: (fill in the blank)

7. Whether the following situations occurred after the COVID-19 infection

Pneumonia (diagnosed by a doctor)

Myocarditis (diagnosed by a doctor)

Respiratory failure requiring mechanical ventilation

Death

No above cases occurred

8. Date of death

9. What is the cause of death diagnosed by a doctor?

10. Have you been hospitalized due to the COVID-19 infection

No, home treatment

Yes, inpatient (general ward)

Yes, hospitalization (intensive care unit)

11. Did you receive the antiviral therapy after infection

No, the following antivirals were not used

Yes, with nematevir\ritonavir (Paxlovid)

Yes, used azvudine

Yes, used monoravir

Other antiviral drugs:

12. Are there any coronavirus sequelae currently?

NO

Yes, please briefly describe the symptoms

13. Whether immunosuppressants are taken regularly before infection

Definition of regular medication: continuous use of the following drugs for more than 3 months, including glucocorticoids (prednisone acetate, etc.), azathioprine, tacrolimus, secopine, Xiaoxi, cyclosporine, methotrexate, cyclophosphamide and other immunosuppressants; Rituximab within 3 months

Not taking medication, or not meeting regular medication definition

Yes, took immunosuppressants regularly

14. Whether the above medication (hormones and immunosuppressants) is suspended due to COVID-19 infection

No, treatment was not interrupted

Yes, self-discontinue the hormone

Yes, self-discontinue immunosuppressants

15. Does myasthenia gravis recur after the infection? If you are not infected with the new crown, has myasthenia gravis recurred since your last visit to the West China Department of Neurology?

NO

YES

16. The specific date of the recurrence

17. What are your symptoms of this recurrence (multiple choices are available)

Ptosis (unilateral or bilateral)

Sight double shadow inarticulate

Difficulty chewing (weak chewing of rice, peanuts, etc.)

Cough when swallowing or drinking water

Weakness of the extremities (upper or lower extremities)

Weakness in neck lifting

Difficulty breathing (wheeze after activity)

Other discomfort: (fill in the blank)

18. The extent of this recurring eyelid ptosis

Not every day

Occurs daily but does not last

Persistent

19. The degree of this recurrence of visual double opacity

Not every day

Occurs daily but does not last

Persistent

20. The extent of slurred speech in this recurrence

Intermittent slurred or nasal sounds

Persistent slurred or nasal sound, audible clearly

Speech that cannot be heard

21. The degree of difficulty in chewing this relapse

Hard food fatigue

Soft food fatigue

Use a gastric tube

22. The degree of recurrent swallowing difficulties or choking on drinking water

Rarely, choking or difficulty swallowing occurs

Dysphagia or dietary changes often occur

Use a gastric tube

23. The degree of limb weakness in this relapse

Standing up in a sitting position sometimes requires upper limb assistance

Standing up in a sitting position usually requires upper limb assistance

Sitting and standing up requires assistance

24. The degree of fatigue in this recurrent neck lift

Brushing your teeth or combing your hair does not require intermittent breaks

Brushing your teeth or combing your hair requires intermittent breaks

Brushing your teeth or combing your hair can't be done on your own

25. The degree of dyspnea this relapse

Shortness of breath during activity

Shortness of breath even when quiet

Use a ventilator

26. Treatment plan selected after recurrence of myasthenia gravis

No special treatment, maintain the original treatment

Adjust the dose of the original oral drug

Add new oral medications (e.g., pyridostigmine bromide, hormones, tacrolimus, azathioprine, etc.)

Intravenous gamma globulin

Plasma exchange

Other, please briefly specify:

27. Whether it has recovered to the state before this recurrence

Yes, it has recovered

No, it has not recovered
