# Supplementary figures and images for: The Risk of Exacerbation of Myasthenia Gravis After COVID‐19 Omicron Infection
Source: Brain Behav. 2024 Oct 20;14(10):e70074. doi: 10.1002/brb3.70074 (PMC11491296; doi:10.1002/brb3.70074)

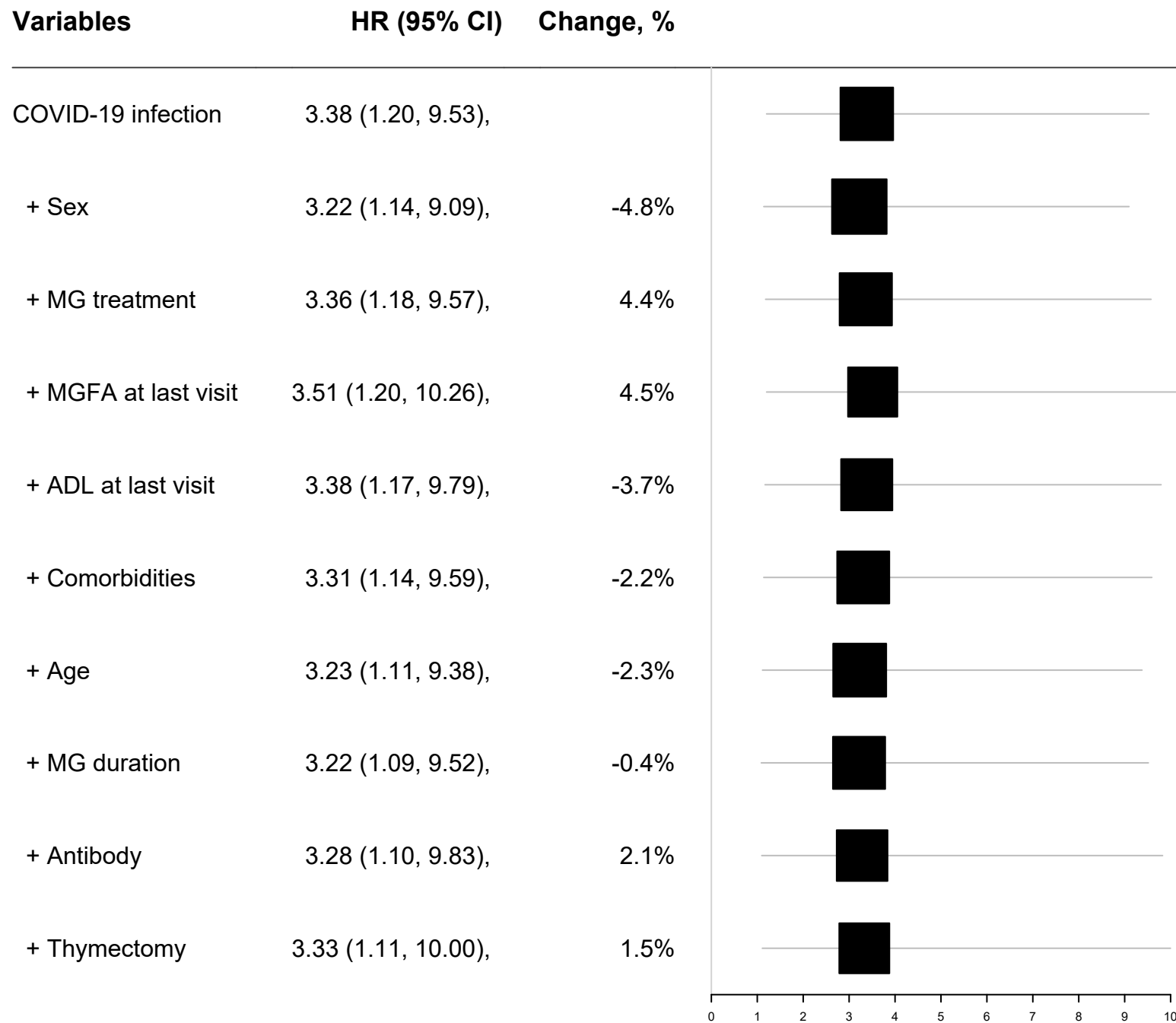

Supplement: Supplementary file 2 — FIGURE S1 Change‐in‐Estimate result. [file BRB3-14-e70074-s003.pdf]

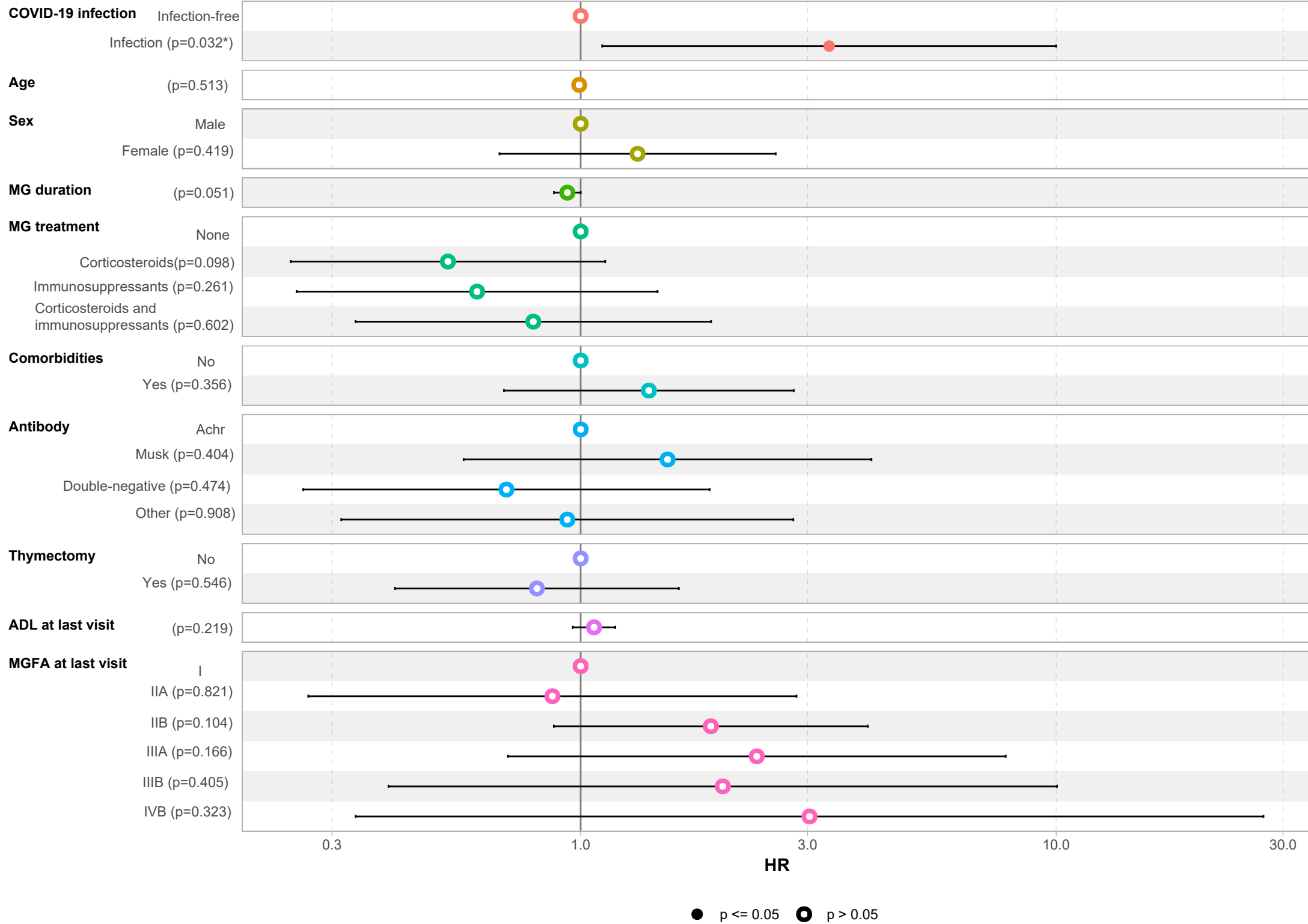

Supplement: Supplementary file 3 — FIGURE S2 The multivariate Cox regression analysis result. [file BRB3-14-e70074-s002.pdf]
