# Supplementary material for: The Risk of Exacerbation of Myasthenia Gravis After COVID‐19 Omicron Infection
Source: Brain Behav. 2024 Oct 20;14(10):e70074. doi: 10.1002/brb3.70074 (PMC11491296; doi:10.1002/brb3.70074)
Supplement: Supplementary file 4 — TABLE S1. The treatment of 20 JTA21 patients. [file BRB3-14-e70074-s005.docx]

| **Supplementary Table 1. The treatment of 204 JTA21 patients** | | | | | |  |
| --- | --- | --- | --- | --- | --- | --- |
|  |  | Treatment in DTM23 | | | | p-value^1^ |
|  |  | 0 | 1 | 2 | 3 |  |
| Treatment in JTA21 | 0 | 21 | 8 | 3 | 3 |  |
|  | 1 | 24 | 54 | 1 | 6 |  |
|  | 2 | 2 | 3 | 41 | 1 |  |
|  | 3 | 4 | 2 | 2 | 29 | 0.07 |
| 1 McNemar-Bowker test | | |  |  |  |  |
