# Supplementary material for: The Risk of Exacerbation of Myasthenia Gravis After COVID‐19 Omicron Infection
Source: Brain Behav. 2024 Oct 20;14(10):e70074. doi: 10.1002/brb3.70074 (PMC11491296; doi:10.1002/brb3.70074)
Supplement: Supplementary file 5 — TABLE S2 The JTA21 patients whose treatment is constant. [file BRB3-14-e70074-s004.docx]

| **Supplementary Table 2. the JTA21 patients whose treatment is constant** | | | | |
| --- | --- | --- | --- | --- |
|  |  | DTM23 | | p-value^1^ |
|  |  | Exacerbation | No exacerbation |  |
| JTA21 | Exacerbation | 0 | 4 |  |
|  | No exacerbation | 23 | 118 | ＜0.001* |
| 1 McNemar’s test | |  |  |  |
| *p < 0.05 was statistically significant | |  |  |  |
